# Assessment of the safety of *Chlorella fusca* grown in refined swine manure liquid fertilizer for bioresource applications

**DOI:** 10.5713/ab.25.0478

**Published:** 2025-09-30

**Authors:** Seukchan Kim, Soo-Ryang Kim, Jungho Moon, Ji-won Jung, Sungha Hong, Sun-Goo Hwang, Myung-Gyu Lee, Meejung Ahn

**Affiliations:** 1 Department of Agricultural Convergence, College of Life and Environment, Sangji University, Wonju, Korea; 2 Industry-Academic Cooperation Foundation, Sangji University, Wonju, Korea; 3 Department of Smart Farm Life Sciences Division, Sangji University, Wonju, Korea; 4 Department of Anatomy and Cell Biology, College of Medicine, Chungnam National University, Daejeon, Korea

**Keywords:** Bioresource, *Chlorella fusca*, Microalgae Medium, Refined Liquid Fertilizer, Safety

## Abstract

**Objective:**

*Chlorella fusca*, a microalga with promising applications in sustainable biotechnology, is of growing interest for its nutritional value, environmental benefits, and bioactive properties. This study investigated the safety and viability of cultivating *C. fusca* in refined liquid fertilizer derived from pig manure.

**Methods:**

Refined liquid fertilizer derived from swine manure was used as a nutrient medium for cultivating *C. fusca*. To compare its biochemical properties with those of commercial *Chlorella* products, the cultivated algae were subjected to proximate composition analyses to determine their water, crude-protein, crude-fat, and ash-free-extract contents. Cytotoxicity was assessed via MTT and WST-1 assays, and an acute toxicity study was performed in Sprague–Dawley rats to evaluate physiological effects according to body weight changes and serum biomarkers. Histopathological examination of the liver, kidneys, and lungs was conducted to detect any toxicological effects. Additionally, antibiotic residues, microbial safety, pesticide contamination, and heavy-metal content were evaluated to confirm overall product safety.

**Results:**

Proximate analysis revealed significant differences in moisture and crude-fat content compared to commercial *Chlorella* products. Cytotoxicity assays demonstrated enhanced immune cell activity at higher concentrations (p<0.05). No significant body weight changes were detected in rats, and serum analysis indicated a dose-dependent reduction in alkaline phosphatase levels in 1,000- and 2,000-mg/mL treatment groups (p<0.05 and p<0.01, respectively). Histopathological examination confirmed the absence of significant alterations in the liver, kidneys, and lungs, supporting its safety as a dietary supplement. Antibiotic residue, microbial safety, pesticide contamination, and heavy-metal analyses showed levels that were undetectable or below safety limits, confirming the safety of *C. fusca*.

**Conclusion:**

*C. fusca* cultivated with refined liquid fertilizer derived from swine manure was found to be safe for use in various biotechnology applications. Our findings imply its potential as a bioresource, including as a feed additive.

## INTRODUCTION

Swine manure is an effective fertilizer that significantly enhances both agricultural productivity and environmental sustainability [1]. Due to its high nutrient levels, gradual nutrient release, and abundance of micronutrients, swine manure significantly enhances plant growth and soil fertility [2]. Livestock manure application is an important strategy for achieving environmentally friendly, resource-circulating agriculture; however, its excessive or inefficient use can lead to serious environmental pollution and economic losses [3]. The potential conversion of livestock manure into value-added products is fundamentally important for advancing sustainable agriculture and mitigating environmental impacts through resource recovery, potentially addressing waste-management challenges and enhancing economic viability through the production of biofertilizers, biofuels, and other functional materials [4].

Microalgae represent a promising renewable nutrition source, leading to growing interest in dietary supplements made from whole-biomass sources such as *Chlorella* and *Arthrospira* species or purified extracts rich in omega-3 fatty acids and carotenoids [5]. The microalga *Chlorella fusca* has shown potential in sustainable biotechnology, particularly in agricultural, nutritional, and environmental applications [1,6]. The *C. fusca* strain ‘CHK0059’ was found to promote the overall growth and performance of strawberry plants [7]. Nanofiber renewal in the cultivation of *C. fusca* ‘LEB 111’ has been demonstrated to enhance CO_2_ biofixation, thereby contributing to reducing atmospheric concentrations of this key greenhouse gas [8].

Our previous studies have focused on the quality control of livestock-manure-based liquid-fertilizer products, particularly with respect to determining key grading factors for a liquid-fertilizer quality certification system [9] and a liquid-fertilizer germination index [10] for use in livestock-manure management. Based on these methodologies, we developed a nutrient medium derived from refined swine-manure-based liquid fertilizer as an alternative to conventional chemical media; its application in *C. fusca* cultivation resulted in higher ascorbic acid and crude-protein levels in bell peppers than in those treated with mineral fertilizers [11]. While cultivated *Chlorella* has previously been evaluated for its effectiveness in agricultural applications, its suitability as a bioresource—such as a feed additive—has not been thoroughly assessed. In this study, we evaluated the safety of cultivated *Chlorella* biomass through acute toxicity testing, nutritional analysis, and regulatory compliance assessment as a preliminary step toward its potential use in biofunctional applications.

## MATERIALS AND METHODS

### *Chlorella* cultivation

**Figure 1:**
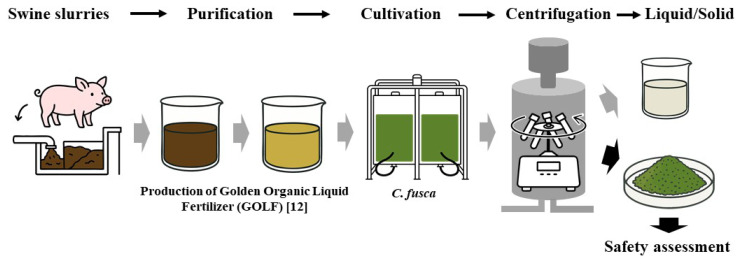
Stepwise schematic diagram of the production process of refined liquid fertilizer derived from swine manure, the cultivation of *Chlorella fusca* in the liquid fertilizer medium, and the subsequent biomass solid production process.

Livestock manure was transformed into an organic cultivation medium based on a previously established method [12]. To prepare the medium for cultivation of *C. fusca*, fermented manure complying with livestock-manure liquid-fertilizer quality certification standards was subjected to electrocoagulation to eliminate suspended solids and enhance optical clarity, making it suitable for algal growth. *C. fusca* strain ‘CHK0059’ was obtained from the National Institute of Agricultural Sciences and grown in an incubator maintained at an average temperature of 28°C until a cell concentration of 1.0×10^7^ cells/mL was reached. Illumination during the cultivation phase was provided by modular light-emitting diodes (FNB-240LED; Nature F&B) that alternated between red and blue wavelengths, under a 16-h/8-h light/dark cycle. Continuous aeration was maintained at a rate of 0.1 m^3^ of air per m^3^ of culture per minute [11]. After sufficient growth, the algal suspension was processed using a tubular continuous centrifuge (J-1050A; Hanil Sci-Med) at 12,000×g to separate the biomass and liquid biofertilizer product. The solid biomass was dried at 45°C in an oven (MOV-212S; Sanyo Electric) for 48 h and pulverized for use in experiments (Figure 1).

### Proximate analysis

The proximate composition (moisture, ash, crude protein, and crude fat) of *C. fusca* was determined following the official AOAC methods: moisture (AOAC 934.01), ash (AOAC 942.05), crude protein (AOAC 984.13), and crude fat (AOAC 920.39) using methods recommended by the Association of Official Analytical Chemists [13].

Moisture content was determined using a moisture balance. Approximately 3.0 g of sample was placed in a pre-dried weighing dish and dried at 105°C in an oven (MOV-212S; Sanyo Electric) for 18 h. The dish was then transferred to a desiccator for 12 h before weighing.

Ash content was measured by dry ashing. Each sample (2 g) was placed in a pre-dried crucible and incinerated in a muffle furnace at 550°C (J-FM3; Jisco) for 5 h. The crucible was then cooled to <100°C and placed in a vacuum desiccator for 3 h before weighing.

Crude-protein content was determined using the semi-micro Kjeldahl method. Each 0.5-g sample was mixed with 10 g of catalyst mixture (9 g K_2_SO_4_ and 1 g CuSO_4_) and 25 mL of concentrated H_2_SO_4_ in a Kjeldahl flask. The mixture was digested by heating until the solution became clear. After cooling, 200 mL of distilled water was added. The mixture was distilled into an Erlenmeyer flask containing 50 mL of 4% boric acid solution and 0.35 mL of indicator (0.1 g methyl red+0.5 g bromocresol green in 0.2% w/v solution). Prior to distillation, 50% NaOH solution and two zinc chips were added to the digestion mixture. The distillate was titrated with 0.1 N HCl to determine the nitrogen content.

Crude-fat content was determined by Soxhlet extraction. Each sample (1 g) was placed in a thimble and extracted with diethyl ether for approximately 10 h at a rate of 4–5 drops/s. After extraction, the ether was evaporated under a fume hood. The thimble was then dried in an oven (MOV-212S; SANYO Electric) at 105°C for 4 h before weighing.

### *Chlorella* powder analysis

Analyses for antibiotic residues, heavy metals, microbial safety, and pesticide contamination were conducted by a certified testing laboratory (JEIL Lab Service) according to the official Korean Food Code (Ministry of Food and Drug Safety, Korea). Specifically, antibiotic residues were tested using liquid chromatography–mass spectrometry following the Korean Food Code guideline; heavy metals were analyzed by inductively coupled plasma mass spectrometry; microbial safety tests were performed using standard plate count and coliform group assays; and pesticide contamination was examined using gas chromatography–mass spectrometry.

For comparative analysis, a commercially available Chlorella powder (Organic Chlorella powder, Nutricost) was used as a reference. The commercial product was stored and handled under the same conditions as the experimental Chlorella powder prior to analysis.

### Cell culture

Mouse T cell lymphoma EL4 cells were cultured in RPMI-1640 medium (RPMI 1640, 11875-093; Thermo Fisher Scientific) supplemented with 10% fetal bovine serum (FBS; 16000-044; Thermo Fisher Scientific), 1% penicillin-streptomycin (15140-122; Thermo Fisher Scientific), and 2 mM L-glutamine (Gibco, Cat. No. 25030-081; Thermo Fisher Scientific). Cells were maintained at 37°C in a humidified atmosphere containing 5% CO_2_ and were used for experiments between passages 8 and 17.

### Cytotoxicity test

MTT and WST-1 assays were performed on cells using assay kits (Abcam) according to experimental protocols recommended by the manufacturer. For the MTT assay, absorbance was measured at wavelengths of 590 nm for the MTT assay and 450–650 nm for the WST-1 assay using a microplate spectrophotometer (LTEK).

### Acute toxicity study

We purchased 25 female Sprague–Dawley rats (6–7 weeks old; 160–200 g) from Daehan Biolink and housed them at the Sangji University Animal Center. Animal care and experimentation adhered to the guidelines of the Sangji University Institutional Animal Care and Use Committee (permission no. 2023-08). The animal housing room was maintained at 24±5°C and 55±5% relative humidity, with a 12-h light/dark cycle.

**Figure 2:**
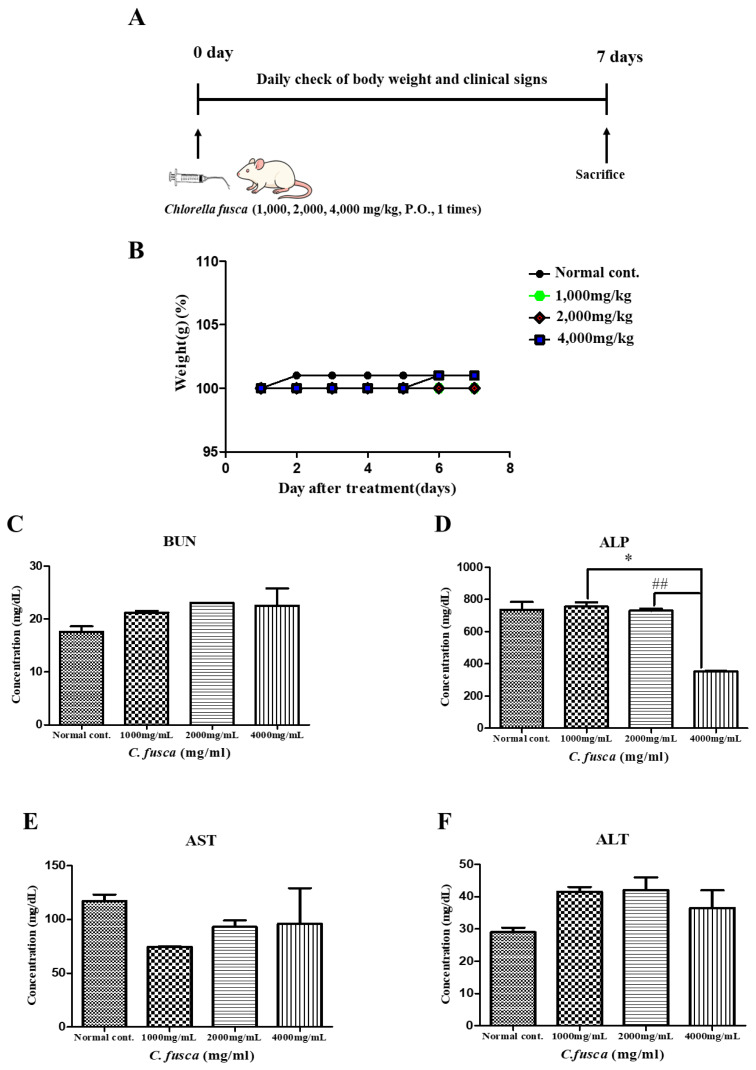
Experimental design used to evaluate the effects of *Chlorella fusca* on acute simple toxicity in rats (A). Changes in body weight during the 7-day experimental period. No significant differences were observed between treatment groups and the normal control group (B). Biochemical markers were analyzed to evaluate the effects of different dosages of the treatment. The markers analyzed include BUN (C), ALP (D), AST (C), and ALT (D). Significant differences were observed: * p<0.05 vs. 1,000 mg/kg treated group, ## p<0.01 vs. 2,000 mg/kg treated group. BUN, blood urea nitrogen; ALP, alkaline phosphatase; AST, aspartate aminotransferase; ALT, alanine aminotransferase.

Following an acclimation period, the rats (n = 25) were randomly assigned to four groups using a completely randomized design. On day 0, the control group (n = 5) and three treatment groups (n = 5 per group) received a single oral gavage dose of *Chlorella* at 0, 1,000, 2,000, or 4,000 mg/kg body weight. Body weight changes were monitored daily for the next 7 days (Figure 2A).

### Tissue sampling

Animals were euthanized on day 7 post-dosing for tissue collection. Anesthesia was induced using isoflurane solution (Hana Pharm). Blood samples were collected for hematological analysis, and liver, lung, and kidney tissues were sampled for histopathological examination. Sampled tissues (liver, lung, and kidney) were fixed in 10% neutral buffered formalin, processed, and embedded in paraffin. For hematological analysis, the levels of alanine aminotransferase (ALT), aspartate aminotransferase (AST), alkaline phosphatase (ALP), and blood urea nitrogen (BUN) were measured. Analytical services were provided by Pureun Clinic.

### Histopathological analysis

Histopathological examination of liver, lung, and kidney tissues was performed using hematoxylin and eosin (H&E) staining. Paraffin-embedded tissue sections (3 μm thick) were prepared using a rotary microtome (Leica), deparaffinized, and stained with hematoxylin for 10 min. After rinsing with distilled water, the sections were counterstained with eosin for 3 min. Following dehydration, clearing, and mounting with Balsam (Daejung), the slides were examined under a light microscope.

### Statistical analysis

All statistical analyses were conducted using GraphPad Prism (ver. 9.5; GraphPad Software). One-way ANOVA was performed to assess overall differences among treatment groups. Post hoc comparisons were conducted using Tukey’s multiple comparisons test. Additionally, orthogonal polynomial contrasts were performed to evaluate linear and quadratic trends in response to increasing Chlorella powder concentrations (0, 1,000, 2,000, and 4,000 mg/kg body weight). Results are expressed as mean±standard deviation (SD), and statistical significance was set at p<0.05.

## RESULTS

### Proximate composition and quality control analysis of *Chlorella* powder

**Figure 3:**
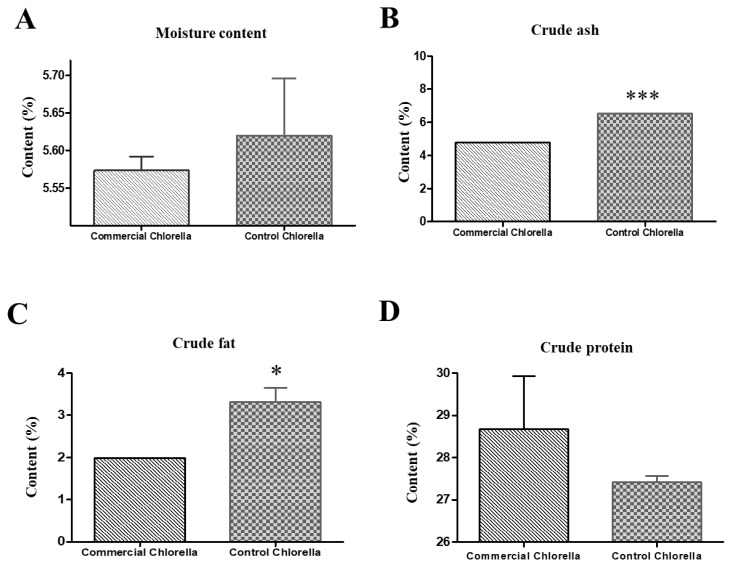
The bar graphs represent (A) moisture, (B) crude ash, (C) crude fat, and (D) crude protein content in the experimental *Chlorella* powder and a commercial product. Significant differences were observed in moisture and crude fat contents between the two groups. Data are presented as mean±standard error of the mean (SEM). * p<0.05, *** p<0.001 vs. commercial product.

The proximate composition of the *Chlorella* powder prepared in this study was compared with that of a commercial *Chlorella* product. Moisture content did not differ significantly between experimental (5.62%) and commercial (5.57%) *Chlorella* samples (Figure 3A). However, ash content was significantly higher in the experimental powder (6.5%) than in the commercial product (4.7%) (p<0.001; Figure 3B), as was crude-fat content (3.32% vs 1.99%) (p<0.05; Figure 3C). Crude-protein content did not differ significantly between the two groups (Figure 3D).

Quality control testing results confirmed that the experimental *Chlorella* powder complied with antibiotic residue, microbial safety, pesticide contamination, and heavy-metal content standards (Table 1).

### Effect of *Chlorella* on cytotoxicity

**Figure 4:**
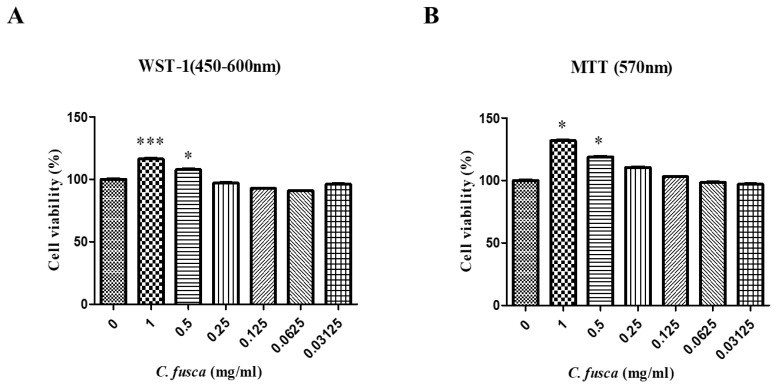
Effects of *Chlorella* powder on cell activity and cytotoxicity. Cell viability and metabolic activity were evaluated using WST-1 (A) and MTT (B) assays after 24-h treatment with increasing concentrations of *Chlorella* powder (0–1 mg/mL). Data are presented as mean±SD. A significant increase in cell activity was observed at concentrations of 0.5 and 1 mg/mL compared to the control group (p<0.05), with no signs of cytotoxicity detected. SD, standard deviation. * p<0.05, *** p<0.001 vs. No treatment.

The cytotoxicity of various concentrations of *Chlorella* powder (0.03125, 0.0625, 0.125, 0.25, 0.5, and 1 mg/mL) compared with an untreated control (0 mg/mL) was assessed using WST-1 and MTT assays. *Chlorella* treatment led to a concentration-dependent increase in cell activity without signs of cytotoxicity. Significant activation was observed only at the highest concentrations (0.5 and 1 mg/mL) (p<0.05; Figure 4).

### Effects of acute *Chlorella* toxicity in rats

The effects of *Chlorella* on liver and kidney function were investigated using biochemical analysis. Body weight was measured over a 7-day period to assess potential toxicity. No significant differences were observed between the treatment groups and the control group. Furthermore, body weight remained stable within all groups compared to their initial values on day 0, indicating that the administration of *Chlorella* powder did not affect overall body weight in the rats (Figure 2B). BUN, ALP, AST, and ALT levels were measured in rat serum. Notably, ALP levels were significantly lower in the 4,000-mg/mL *Chlorella* treatment group than in the 1,000-mg/mL (p<0.05) and 2,000-mg/mL (p<0.01) groups (Figure 2D). There were no significant differences in BUN, AST, or ALT levels among groups (Figures 2C, 2E, 2F).

### Histopathological examination of target organs

**Figure 5:**
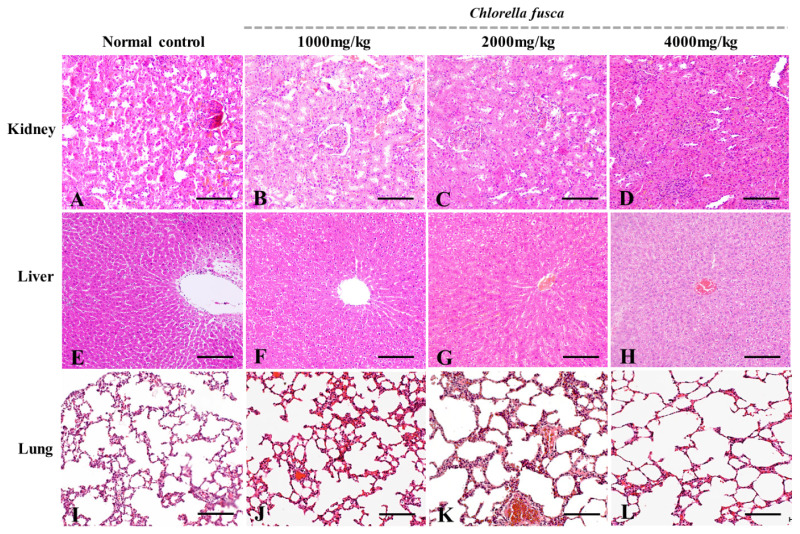
Histological findings of *Chlorella* powder on acute simple toxicity in rats. Kidney (A–D), Liver (E–H), and lung (I–L) were sectioned and stained with H&E staining: (A, E, I) normal control, (B, F, J) 1,000 mg/mL *Chlorella* powder, (C, G, K) 2,000 mg/mL *Chlorella* powder control, (D, H, L) 4,000 mg/mL *Chlorella* powder. Scale bars = 100 μm.

Histopathological examination of kidney, liver, and lung tissues from rats in each group was performed following *Chlorella* treatment at doses of 1,000, 2,000, and 4,000 mg/mL (Figure 5). No histopathological alterations were observed in kidney, liver, or lung tissues from any treatment group compared to the control group. Similarly, no signs of inflammation or tissue damage were detected in any of the examined organs.

## DISCUSSION

The cultivation of *C. fusca* using alternative nutrient sources, such as livestock-manure-derived liquid fertilizers, presents a promising approach to achieving circular bioeconomy goals. High-temperature aerobic liquid fermentation of livestock manure followed by advanced purification processes such as electrocoagulation and ultrafiltration ensures a nutrient-rich, safe growth medium for microalgae cultivation [14,15].

Livestock manure, a major byproduct of intensive animal production systems, possesses agronomic value as a nutrient-rich organic amendment when managed under controlled conditions. However, improper handling, storage, or application can result in adverse environmental outcomes, including eutrophication of aquatic ecosystems due to nutrient runoff, emission of greenhouse gases such as methane and ammonia that contribute to air pollution and climate change, and degradation of soil quality through the accumulation of heavy metals and pathogens [16]. Therefore, sustainable management of livestock manure is a critical component in mitigating the ecological footprint of livestock farming.

The findings of this study provide significant insights into the biochemical composition, health effects, and safety of *C. fusca* as a functional dietary supplement. Cultivation of *C. fusca* using sustainable, alternative nutrient sources has demonstrated its potential for cost-effective, environmentally friendly biomass production [17]. Livestock-manure-derived liquid fertilizer that has been purified through electrocoagulation and ultrafiltration offers a nutrient-rich medium suitable for large-scale microalgal cultivation [18]. Our proximate analysis revealed that the experimental *Chlorella* powder had significantly higher moisture and lipid contents compared to a commercial product, highlighting the substantial impact of cultivation conditions and processing methods on the biochemical composition of such powders [19]. Environmental factors such as light exposure and nutrient availability significantly affect the protein and antioxidant contents of *C. fusca* [20]. Additionally, *Chlorella* contains valuable components that have significant potential for CO_2_ fixation during biomass production, enabling diverse biotechnological applications [15].

Our cytotoxicity assessments, including MTT and WST-1 assays, confirmed the safety of *C. fusca* for cellular applications, supporting its biocompatibility [21]. A previous animal study demonstrated that *C. fusca* supplementation does not induce significant histopathological alterations in vital organs, reinforcing its safety for consumption [17]. Biochemical analysis revealed no significant differences in BUN, ALT, or AST levels compared to the control group, indicating that *C. fusca* does not exhibit acute toxicity in animals. The dose-dependent reduction in ALP levels implies potential hepatoprotective effects, consistent with a previous study that reported liver-supporting properties of *Chlorella* species [19]. While a reduction in ALP was observed, this may reflect various physiological changes rather than definitive hepatoprotective effects. Further studies, including additional liver biomarkers and appropriate disease models, are required to elucidate the potential hepatoprotective properties of *C. fusca*.

Despite these promising findings, this study had some limitations. We used only one animal species; further research involving additional species should be conducted to validate the safety and efficacy of *C. fusca* cultivated using refined liquid fertilizer derived from swine manure [22]. Additionally, long-term toxicity studies are required to evaluate its chronic effects and possible bioaccumulation risks [23]. Future studies should focus on optimizing cultivation techniques to maximize the nutritional and functional compound yield of *C. fusca* [24].

## CONCLUSION

The results of this study provide important evidence supporting the sustainable cultivation of *C. fusca* as a bioresource in applications such as feed additives. This microalga may show potential benefits in foods, environmental sustainability, and functional health products, further enhancing its promise as a safe, functional dietary supplement. The potential conversion of swine manure into a high-value bioresource further underscores the significance of stable biomass production. Based on the 7-day acute toxicity results, *C. fusca* appears to be safe under the tested conditions; however, further chronic toxicity studies are required to fully evaluate its safety for long-term use as a functional dietary supplement. Future research on the bioactive compounds and mechanisms of action identified in this study is expected to contribute to enhancing its utilization for health improvement and as a valuable bioresource.

## Figures and Tables

**Table 1:** Analysis of antibiotics, microbial contaminants, pesticides, and heavy metals in *Chlorella powder*

Category	Specific test item	Result	Acceptance criteria
Antibiotics	Aminoglycoside	Not detected	
	Sulfonamide	Not detected	
	Cephalosporin	Not detected	
	Quinolone	Not detected	
	Penicillin	Not detected	
	Tetracycline	Not detected	
	Phenicol	Not detected	
	Macrolide	Not detected	
	Peptide	Not detected	
	Other antibiotics	Not detected	
Microbiology	*Salmonella*	Negative	
	*Staphylococcus aureus*	Negative	
	*Listeria monocytogenes*	Negative	
	*Clostridium perfringens*	Negative	
	Shiga toxin-producing *Escherichia coli*	Negative	
	*Bacillus cereus*	Negative	
	*Vibrio parahaemolyticus*	Negative	
	Coliform	Negative	
	*E. coli*	Negative	
	Yeast & mold	2,900 CFU/g	
Pesticides	463 Pesticides	Not detected	
Heavy metals	Benzo[a]pyrene	Not detected	
	Cadmium^a^	0.02 mg/kg	2.5 mg/kg
	Lead^b^	2.92 mg/kg	30 mg/kg
	Arsenic^c^	1.81 mg/kg	25 mg/kg

Quantitative analysis demonstrated that the concentrations of ^a, b^, and ^c^ were all below the respective acceptance criteria established by feed ingredient standards.
